# Secondary cardiac valvular disease in neuroendocrine tumour: a case report highlighting echocardiographic features

**DOI:** 10.1093/ehjcr/ytag132

**Published:** 2026-02-25

**Authors:** Xinmin Chen, Jing Yao, Xiaoxian Wang, Aijuan Fang, Mingxia Li

**Affiliations:** Department of Ultrasound Medicine, Nanjing Drum Tower Hospital, The Affiliated Hospital of Nanjing University Medical School, 321 Zhongshan Road, Nanjing 210008, China; Department of Ultrasound Medicine, Nanjing Drum Tower Hospital, The Affiliated Hospital of Nanjing University Medical School, 321 Zhongshan Road, Nanjing 210008, China; Department of Ultrasound Medicine, Nanjing Drum Tower Hospital, The Affiliated Hospital of Nanjing University Medical School, 321 Zhongshan Road, Nanjing 210008, China; Department of Ultrasound Medicine, Nanjing Drum Tower Hospital, The Affiliated Hospital of Nanjing University Medical School, 321 Zhongshan Road, Nanjing 210008, China; Department of Ultrasound Medicine, Nanjing Drum Tower Hospital, The Affiliated Hospital of Nanjing University Medical School, 321 Zhongshan Road, Nanjing 210008, China

## Case description

A 50-year-old woman presented in May 2025 for evaluation of hepatic masses and abnormal liver function (alanine aminotransferase [ALT] 110 U/L, aspartate aminotransferase [AST] 108 U/L), with 2 months of bilateral eyelid oedema. The patient reported significant facial flushing but denied nausea, vomiting, chest pain, palpitations, chest tightness, or dyspnoea. She had no relevant prior medical history or regular medication use.

**Figure 1:**
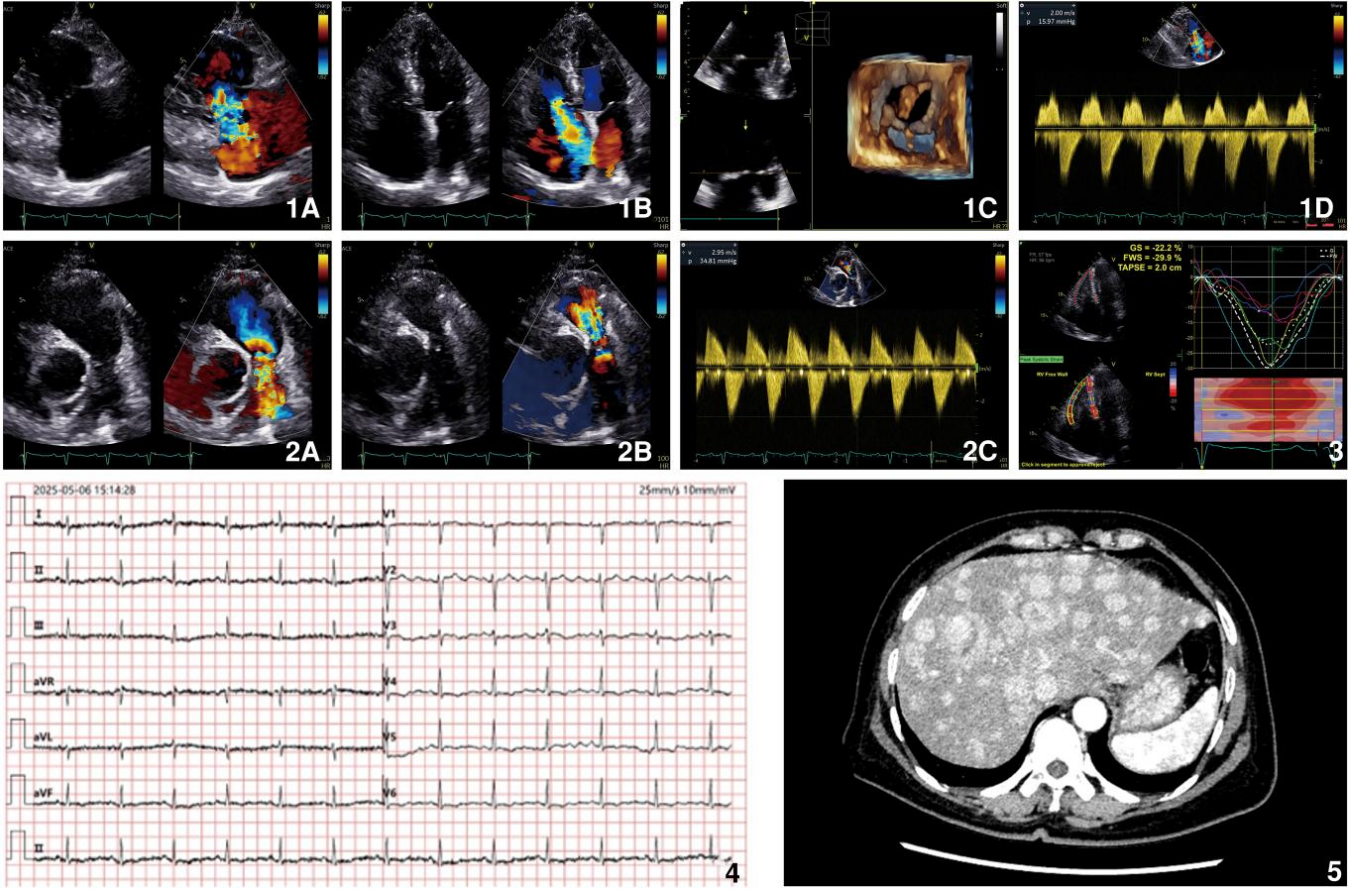
Panels 1 and 2 depicted valvular pathologies: tricuspid valve stenosis/regurgitation with a peak gradient of 16.0 mmHg, and severe pulmonary valve stenosis/regurgitation with a peak systolic gradient of 34.8 mmHg, respectively. Panel 3 presented right ventricular strain results, Panel 4 displayed the ECG, and Panel 5 revealed multiple hepatic space-occupying lesions.

The observed electrocardiogram (ECG) findings-sinus rhythm with ST-T changes and poor R-wave progression-were likely attributable to the patient's hypokalaemia (serum K^+^, 2.9 mmol/L), given the normal troponin T level (*Figure 1*, Panel 4). Transthoracic echocardiography (TTE) revealed normal left ventricular size and systolic function [left ventricular ejection fraction (LVEF), 62.4%], with structurally normal mitral valves. In contrast, significant right heart abnormalities were observed: The tricuspid valve leaflets were thickened, shortened, and retracted (*Figure 1*, Panel 1; Supplementary material online, Videos 1–3), resulting in severe combined stenosis and regurgitation. Similar findings were noted at the pulmonary valve (*Figure 1*, Panel 2; Supplementary material online, Video 4). Right ventricular function was preserved: right ventricular global longitudinal strain (RVGLS), −22.2%; right ventricular free wall strain (RVFWS), −29.9%; and tricuspid annular plane systolic excursion (TAPSE), 2.0 cm (*Figure 1*, Panel 3).

The constellation of eyelid oedema, facial flushing, abnormal liver function with hepatic lesions, and echocardiographic valvular pathology supported a diagnosis of carcinoid heart disease (CaHD). As prior imaging failed to locate the primary tumour (*Figure 1*, Panel 5), liver biopsy confirmed neuroendocrine tumour metastasis. Given the confirmed diagnosis, a multidisciplinary team recommended surgical valve replacement. However, the patient declined all further interventions and was discharged.

CaHD has a poor prognosis, with a three-year survival rate of only 31% in untreated patients.^1^ It affects ∼90% of patients with moderate-to-severe tricuspid regurgitation and 85% with pulmonary valve disease, primarily regurgitation or stenosis.^2^ TTE is the recommended initial imaging modality for diagnosis and monitoring,^3^ with baseline assessment including valvular structure and function, chamber dimensions, and ventricular performance.

## Supplementary Material

ytag132_Supplementary_Data

## Data Availability

Data cannot be shared for ethical/privacy reasons.

## Notes

**Consent:** Informed consent for publication was obtained from the patient in line with COPE guidelines.
